# Primary Signet Ring Cell Carcinoma of Rectum Diagnosed by Boring Biopsy in Combination with Endoscopic Mucosal Resection

**DOI:** 10.1155/2018/5860815

**Published:** 2018-01-10

**Authors:** Yoshito Hirata, Keishi Kanno, Nobusuke Kishikawa, Shinji Tomoda, Kazuki Kimura, Tomoki Kobayashi, Daisuke Miyamori, Yuichiro Otani, Masafumi Mizooka, Koji Arihiro, Shiro Oka, Shinji Tanaka, Susumu Tazuma

**Affiliations:** ^1^Department of General Internal Medicine, Hiroshima University Hospital, 1-2-3 Kasumi, Minami-ku, Hiroshima 734-8551, Japan; ^2^Department of Anatomical Pathology, Hiroshima University Hospital, 1-2-3 Kasumi, Minami-ku, Hiroshima 734-8551, Japan; ^3^Department of Endoscopy, Hiroshima University Hospital, 1-2-3 Kasumi, Minami-ku, Hiroshima 734-8551, Japan

## Abstract

A 46-year-old man with severe back pain visited our hospital. Magnetic resonance imaging revealed extensive bone metastasis and rectal wall thickness. Colonoscopy revealed circumferential stenosis with edematous mucosa, suggesting colon cancer. However, histological findings of biopsy specimens revealed inflammatory cells but no malignant cells. The patient underwent endoscopic ultrasound, which demonstrated edematous wall thickness without destruction of the normal layer structure. After unsuccessful detection of neoplastic cells by boring biopsies, we performed endoscopic mucosal resection followed by boring biopsies that finally revealed signet ring cell carcinoma. Herein, we present a case and provide a review of the literature.

## 1. Introduction

Signet ring cell carcinoma (SRCC) is a subtype of mucinous carcinoma, which is histologically characterized by cancer cells with abundant intracytoplasmic mucin and peripherally pushed nuclei. Majority of SRCCs arise in the stomach, with the rest occurring in other organs including the breasts, gallbladder, pancreas, urinary bladder, and colorectum [1]. Colorectal SRCC, which is rare, with a reported incidence of 0.1%–2.6% of all primary colorectal cancers, is clinicopathologically different from ordinary colorectal adenocarcinomas in terms of early onset, higher rate of peritoneal seeding, lower rate of liver metastasis, higher distant metastasis, and poor prognosis [2]. In addition, SRCC has high propensity for intramucosal spreading with relative sparing of the mucosa. Therefore, cancer cells are rarely detected in some cases in regular biopsies [3, 4]. Here, we describe a case of SRCC of the rectum in which definite diagnosis was achieved by boring biopsies (also known as bite-on-bite biopsies) in combination with endoscopic mucosal resection (EMR).

## 2. Case Report

**Figure 1:**
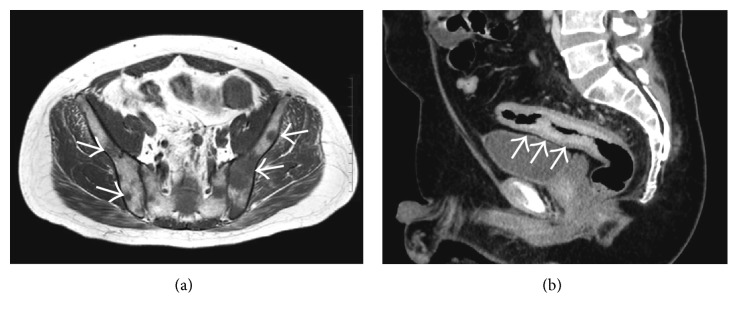
(a) T1-weighted MRI of the pelvis demonstrates multiple lesions in the ilium with low T1 signal intensity (arrows), suggesting multiple bone metastases. (b) Abdominal contrast-enhanced CT scan reveals long segmental bowel thickening in the rectum (arrows).

A 46-year-old man presented to the orthopedic department of our hospital with a 1-week history of pain in rear neck, back, and left hip joint. Magnetic resonance imaging (MRI) revealed low T1 signal intensity spreading diffusely in multiple vertebrae as well as the ilium, suggesting multiple bone metastases (Figure 1(a)). Rectal wall thickness was also found. Therewith, a new onset of abdominal pain was detected; therefore, the patient was referred to our department to determine the primary lesion associated with multiple bone metastases and evaluate the cause of abdominal pain.

On admission, the patient could neither walk nor change his position on bed because of severe pain without paralysis. He had a past history of traumatic injury at the age of 25 and had undergone splenectomy; however, there was no history of inflammatory bowel disease. Moreover, there was no family history of cancer. He had sleep disturbance because of pain and suffered from frequent and watery bowel movement approximately 10 times per day. Abnormalities detected by the laboratory test included leukocytosis (white blood cell count: 16,200/*µ*L) with a high level of C-reactive protein (17.83 mg/dL). There was no anemia (hemoglobin: 16.9 g/dL). Serum alkaline phosphatase (ALP) and lactate dehydrogenase (LDH) levels were 1390 IU/L and 583 IU/L, respectively, which are most likely because of bone metastasis. Tumor markers, such as carcinoembryonic antigen and CA19-9, showed slightly elevated levels (5.4 ng/mL and 55 U/mL, resp.).

**Figure 2:**
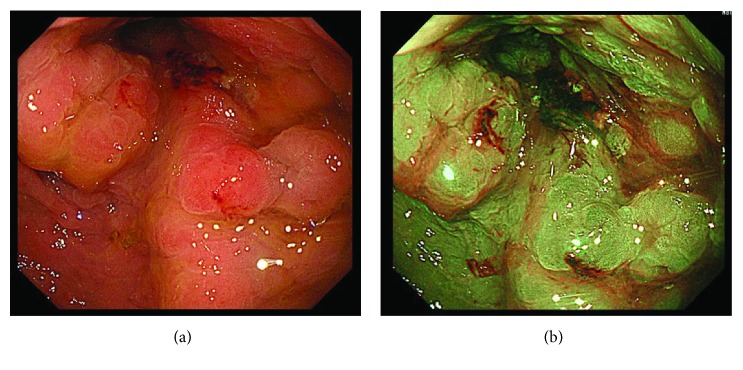
(a) Colonoscopy identifies edematous stenotic lesion spreading over the entire circumference in the rectum at 10–18 cm from the anal verge. (b) Chromoendoscopy using indigo carmine was performed.

**Figure 3:**
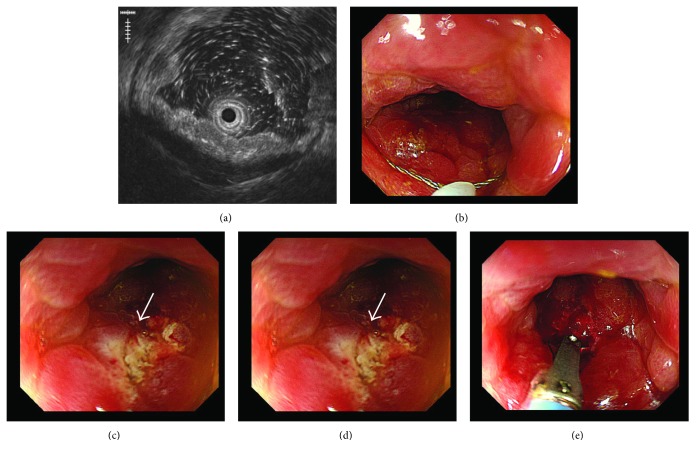
(a) Miniprobe ultrasound shows diffuse thickening of the rectal wall without destruction of a 5-layer structure. (b–d) Endoscopic mucosal resection was performed with a conventional electrosurgical snare to remove the edematous thick mucosa, followed by boring biopsies from the resected area (arrow) to obtain deeper specimens.

**Figure 4:**
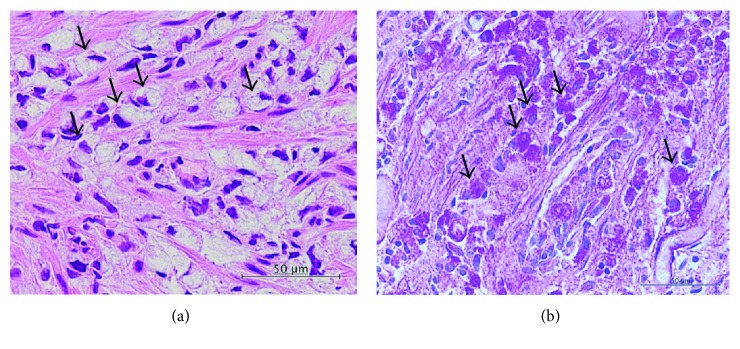
Hematoxylin and eosin (a) and periodic acid-Schiff (b) staining demonstrate scattered SRCC cells (arrows) in the lamina propria mucosae with fibrous stromal reaction.

**Figure 5:**
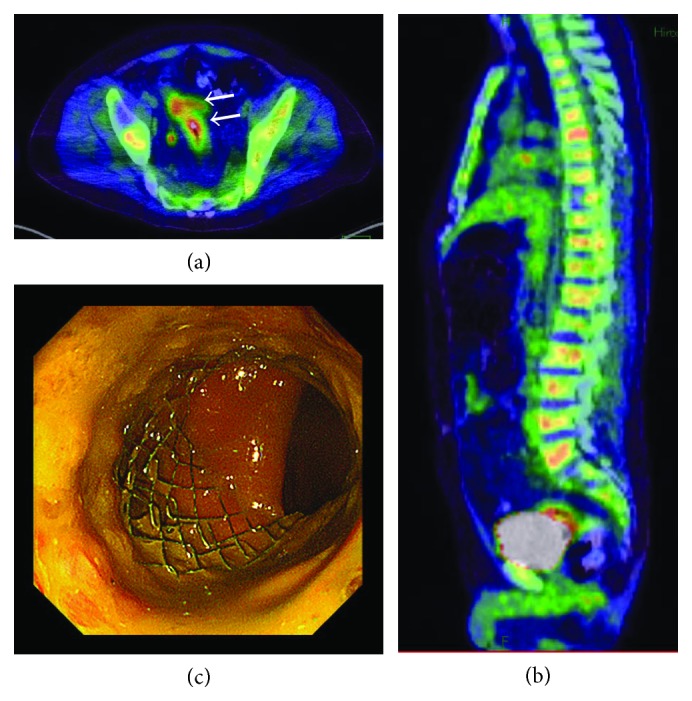
(a and b) CT scanning with positron-emission tomography shows significant FDG uptake in the rectum (arrows) and extensive bone metastasis in the pelvis and vertebrae. (c) The self-expandable metal stent was placed endoscopically.

Computed tomography (CT) scan of the abdomen and pelvis with contrast revealed diffuse concentric thickening of the rectum with narrowing of the lumen and regional lymphadenopathy (Figure 1(b)). Considering the findings of MRI and CT, the rectum was suspected as the primary site of cancer; therefore, colonoscopy was performed at the department of endoscopy. There was edematous lesion spreading over the entire circumference of the rectum at 10–18 cm from the anal verge (Figures 2(a) and 2(b)). Pathological examination of multiple biopsy specimens demonstrated chronic inflammation with no malignant cells. On another day, endoscopic ultrasound (EUS) was performed to assess the rectal lesion, which revealed diffuse thickening of the colonic lumen without destruction of the normal layer structure (Figure 3(a)). In addition, we performed biopsies with each bite directly on the top of the previous bite to obtain a deeper tissue sample for histological diagnosis. This biopsy procedure is called as boring biopsy or bite-on-bite technique. However, none of the specimens confirmed malignancy. Upper endoscopic examination revealed no primary lesions. To obtain sufficient tissue samples from deeper layers, we next performed boring biopsy in combination with EMR. In brief, we removed overlying edematous epithelia using an electric snare without mucosal lift by injection and then carried out boring biopsies from the resected area (Figures 3(b)–3(d)). Histological examination found irregularly scattered SRCC cells in the lamina propria mucosae (Figure 4), which confirmed rectal SRCC with multiple bone metastases (stage IVB). CT scanning with positron-emission tomography (PET-CT) revealed extensive bone metastasis with significant uptake of FDG in the rectum (Figures 5(a) and 5(b)). Considering poor performance status and widespread metastasis, the patient underwent radiotherapy with a total dose of 50 Gy to reduce back pain and stent placement to release colon obstruction (Figure 5(c)). Regardless of the treatment with multiple opioids and zoledronic acid, his performance status remained poor due to pain with concomitant systemic inflammation of the undetermined origin. Under such a condition, the informed patient chose to have just symptom control rather than invasive treatments and was transferred to a palliative care unit in another hospital for best supportive care.

## 3. Discussion

It has been reported that more than 96% of the SRCCs arise in the stomach. Colorectal SRCC is a very rare subtype, which accounts for only 0.1%–2.6% of all colorectal cancer cases [2]. In addition to its rarity, colorectal SRCC is considered to have poor prognosis as compared to conventional adenocarcinomas because of its high propensity for diffuse intramural infiltration, lymph node involvement, peritoneal dissemination, and distant metastasis [5]. In fact, previous age- and sex-matched controlled study has demonstrated that the survival rate of patients with SRCC was significantly lower than that of ordinary adenocarcinomas with independent predictive factors, such as the stage of diagnosis and presence of distant metastasis [6]. In agreement, the patient in this case visited the hospital because of back and neck pain caused by multiple bone metastases without severe digestive organ symptoms and was diagnosed with the advanced stage of SRCC (Stage IVB).

Based on the finding of CT and colonoscopy, diffuse infiltrative carcinoma of the rectum was strongly suspected; however, neither regular biopsies nor boring biopsies detected cancer cells. Similar cases of SRCC have been previously reported [4, 5]. In these cases, many biopsy specimens were unable to identify malignant cells, and subsequent biopsy from the erosive lesion finally led to a definitive histological diagnosis. In our case, there were no affected regions in the main stenotic lesion, likely suggesting that cancerous lesion was covered with normal epithelia. Following the negative results for malignancy by conventional biopsies as well as boring biopsies, we performed boring biopsy in combination with EMR to obtain sufficient specimens from deeper layer, and finally, definitive diagnosis was achieved. In this regard, several methods have been previously proposed to obtain the tissues from subepithelial lesion. These include jumbo biopsy using large-capacity forceps, EUS-guided fine-needle aspiration, and EMR [7]. In addition, there have been reports demonstrating the advantage of tissue sampling using the bite-on-bite technique with or without EMR in esophagogastroduodenal subepithelial lesions and infiltrating gastric tumors [8–10], collectively suggesting that multiple deep biopsies are required for effective diagnosis. Boring biopsies in combination with EMR performed in this case may be a novel tissue acquisition method for the pathological confirmation of colorectal SRCCs when regular biopsies show negative results.

In this case, metastatic bone tumor was pointed out before the identification of primary lesion of rectal cancer. According to previous reports, the prevalence of bone metastasis from colorectal cancer is 8.6%–27% in autopsy cases and 3.7%–11% in clinical cases [11, 12]. With respect to the location, the rectum is the most frequent, with a reported incidence of 32.4%–46.8% in cases of bone metastasis from colorectal cancers [11, 12]. Considering that the patient had no liver or lung metastasis, the delivery route is unlikely via systemic blood circulation; in contrast, the route is more likely via vertebral venous plexus that has communications with the pelvis and vertebral bodies, as described by Batson [13].

In summary, we herein report a case of rectal SRCC diagnosed by boring biopsies in combination with EMR. To the best of our knowledge, this is the first case report that demonstrates the usefulness of this deep biopsy technique for achieving diagnosis of rectal infiltrating tumors. This diagnostic method should be considered when ordinary endoscopic biopsy results are negative for cancer cells.
